# Complete Genome Sequences of the Plasmid-Bearing *Campylobacter coli* Strains HC2-48, CF2-75, and CO2-160 Isolated from Retail Beef Liver

**DOI:** 10.1128/genomeA.01004-16

**Published:** 2016-09-15

**Authors:** Daya Marasini, Mohamed K. Fakhr

**Affiliations:** Department of Biological Science, The University of Tulsa, Tulsa, Oklahoma, USA

## Abstract

The complete genome sequences of *Campylobacter coli* strains HC2-48, CF2-75, and CO2-160, isolated from retail beef liver, showed the presence of 1,663,782-, 1,711,393-, and 1,683,224-bp circular chromosomes and 44,064-, 44,233-, and 44,228-bp circular plasmids, respectively. This is the first reported *Campylobacter coli* genome sequence isolated from retail beef liver.

## GENOME ANNOUNCEMENT

While most *Campylobacter* foodborne illnesses are caused by *C. jejuni*, around 7% are associated with *C. coli* (1). *C. coli* is more prevalent in retail beef liver and shows higher multidrug resistance compared to *C. jejuni* (2). In a recent study, the prevalence of mega plasmids above 100 kb in size was higher in *C. coli* than in *C. jejuni* strains isolated from various retail meats (3). The genome size of *C. coli* usually ranges in size between 1.6 Mb and 1.7 Mb (4–6). To our knowledge, only one retail beef liver *C. jejuni* genome was reported for strain YH001 (7). Hence, we announce here the first complete genome sequence of three *C. coli* strains isolated from retail beef liver.

The total genomic DNA was isolated using the DNeasy blood and tissue kit (Qiagen Inc., Valencia, CA, USA) from a 72-h culture grown microaerobically in Mueller-Hinton blood media. The complete genome sequencing was performed by a MiSeq desktop sequencer using a paired-end 2 × 150-cycle V2 reagent kit from Illumina (Illumina Inc., San Diego, CA, USA). The DNA library preparation was done using the Nextera XT library preparation kit (Illumina Inc.). The reads were assembled using CLC Genomics Workbench version 7.5.1, and the contigs were arranged using the Microbial Genome Finishing Module version 1.4 available in CLC Genomics Workbench (Qiagen Inc.) against the closest reference genome.

The complete genome sequence of the *C. coli* isolates HC2-48, CF2-75, and CO2-160 revealed the presence of one circular chromosome and one circular plasmid in each of the strains. The chromosome sizes of strains HC2-48, CF2-75, and CO2-160 were 1,663,782 bp, 1,711,393 bp, and 1,683,224 bp, respectively. Plasmids harbored by strains HC2-48, CF2-75, and CO2-160 were 44,064 bp, 44,233 bp, and 44,228 bp in size, respectively. There were a total of 1,767 genes containing 1,667 coding sequences (CDSs), 46 pseudogenes, and 54 RNAs present in the genome of HC2-48, out of which 46 genes were present on the plasmid. This plasmid showed the presence of tetracycline resistance (*tet*M) and aminoglycoside phosphor-transferase genes. The genome of strain CF-75 revealed the presence of 1,840 genes containing 1,742 CDSs, 44 pseudogenes, and 54 RNAs, while its plasmid contained 44 genes, including the tetracycline resistance protein (*tet*M) gene, along with two pseudogenes. The genome of strain CO2-160 contained 1,790 genes with 1,697 CDSs, 39 pseudogenes, and 54 RNAs, while the plasmid showed the presence of 47 genes, including the tetracycline resistance gene (*tet*M) found on the other two plasmids reported in this study. All three plasmids also contained Cag pathogenicity island proteins and type IV secretion system. The chromosome sequences of all three *C. coli* retail beef liver strains were quite similar and were closer in sequence homology to the *C. coli* isolate RM5611.

### Accession number(s).

The GenBank accession numbers of the chromosomes and plasmids of the *C. coli* isolates sequenced in this study are as follows: HC2-48 (CP013034 and CP013035), CF2-75 (CP013036 and CP013037), and CO2-160 (CP013032 and CP013033).
